# Application of cook balloon during aorta replacement in a pregnant Marfan-syndrome patient: a case report

**DOI:** 10.1186/s12884-020-02871-6

**Published:** 2020-03-18

**Authors:** Lu Zhang, Bin Yan, Xue Cui, Jinghong Liu, Fangxin Shi

**Affiliations:** 1 Department of Obstetrics and Gynecology, First Affiliated Hospital of Dalian Medical University, Dalian, 116011 China; 2 Department of Radiology, First Affiliated Hospital of Dalian Medical University, Dalian, China

**Keywords:** Aortic dissection, Marfan syndrome, Pregnancy, Cook balloon

## Abstract

**Background:**

Aortic dissection is a rare and emergent condition. Aortic dissection during pregnancy is not much known but it is quite lethal to both mother and infant. Earlier reports published show that clinicians conducted hysterectomies during cesarean section to avoid anticoagulant-induced uterine bleeding during the following aortic surgery.

**Case presentation:**

A woman (38, gravida 1, para 0) in the 37th gestational week suffered an acute, severe, sharp pain in the chest and back. She was diagnosed with Standford type A aortic dissection and suspected with Marfan syndrome. An emergency cesarean section was performed immediately to deliver the baby. Since the patient was on anticoagulants during aortic replacement, so Cook balloon was inserted into the uterus to prevent postpartum hemorrhage. This helped to maintain the uterus intact. Family genetic testing showed that the patient was a carrier of FBN1 mutation which was inherited from the patient’s mother, and the newborn also carried the mutation. Hence the patient was concluded to be positive for Marfan syndrome.

**Conclusion:**

It is important that clinicians should pay attention to the possibility of aortic dissection in a pregnant woman with chest, abdominal or back pain. In this case study, we employed Cook balloon during cesarean section to avoid anticoagulant-induced uterine bleeding during the following aortic surgery.

## Background

Aortic dissection is a relatively rare condition, with a general occurrence rate of 2.9/100,000/yr [1]. It is, therefore, crucial to diagnose the condition correctly and rapidly and to administer immediate and appropriate treatment. Aortic dissection during pregnancy is even rarer than in normal patients. Pregnancy seems to increase the risk of aortic dissection in women with Marfan syndrome on account of blood-vessel alterations, particularly in the third trimester [2]. Through this study, we report successful treatment of Standford type A aortic dissection in a pregnant Marfan-syndrome patient.

## Case presentation

**Fig. 1:**
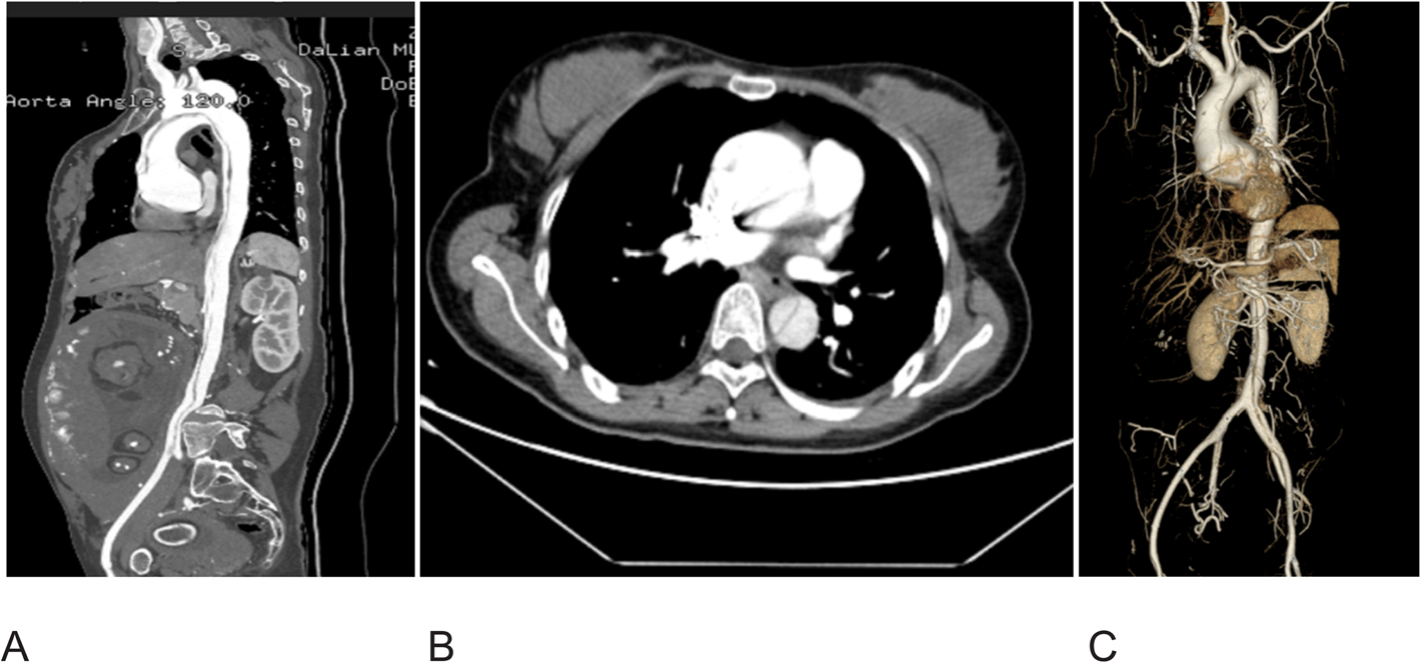
Aortic computed tomography angiograms. **a** Aortic dissection from ascending aorta to abdominal aorta and the fetus in the uterine cavity. **b** Acute aortic dissection with a false lumen. **c** 3-D reconstruction of the aorta, showing dilated aortic sinuses of Valsalva

The patient at hand was a woman (38, gravida 1, para 0) in the 37th gestational week. She suffered an acute, severe, sharp pain in the chest and back and was admitted to our hospital in January 2018. She had no history of vascular disease or hypertension and was uncertain of familial history. Her blood pressure measured 107/38 mmHg. A transthoracic two-dimensional echocardiography revealed a severely dilated aortic root (Ø 58 mm) and severe aortic valve insufficiency. Aortic computed tomography angiography (CTA) led to the diagnosis of the Standford type A aortic dissection (Fig. 1). The patient was slender with a height of 173 cm and featured spider-like fingers and toes (having “wrist sign”). We suspected her having Marfan syndrome. The fetal ultrasound was inconspicuous, with a normally developed fetus and 3 h post-admission, the patient underwent an emergency cesarean section with full anesthesia. Blood pressure was carefully monitored during the surgery. A 2950-g infant was born (Apgar score: 9 at 1 min, 10 at 5 min). After the baby was born, the patient was administered with 20u oxytocin intravenously. To prevent postpartum bleeding during aorta replacement, potentially caused by the anticoagulants in the extracorporeal circulation, we inserted Cook balloon containing 400 ml of normal saline into the uterus during the cesarean section. The aortic root, ascending aorta and aortic arch was replaced under cardiopulmonary bypass and hypothermia (25 °C). Vaginal bleeding during the six-hour surgery was modest (< 150 ml). The Cook balloon was removed from the patient 24 h post-surgery leaving an intact uterus. The patient was released after 20 days. In order to make a definite diagnosis, we did the genetic testing of the patient’s family. The patient carried an FBN1 mutation and the site was chr15:48905206. The mutation was NM_000138.4:c.247 + 1G>T. We also discerned that the patient’s Z score of aortic root diameter was 7.5. The above findings were correlated to the Ghent-2 criteria and we confirmed that she was having Marfan syndrome. Family genetic testing showed that the mutation inherited from the patient’s mother, and the newborn also carried the mutation. We explained the result of genetic testing to the patient and the genetic mode of autosomal dominant inheritance. She was advised contraception and restricting activity. Also we made her aware of the impact Marfan syndrome would have on her newborn’s health. Both she and her baby need a close follow-up. The patient was followed up by the cardiologist three times after discharge.

## Discussion and conclusion

Marfan syndrome (MFS) is a dominant, autosomal inherited disorder of connective tissue that leads to the damage of cardiovascular, skeletal and ocular systems. The most serious MFS-induced complication is aortic dissection. The overall risk of a pregnant woman with MFS having an aortic dissection is about 3% [3] wherein dissection occurs often either in the last trimester of pregnancy or the early postpartum period. A recent study comprising of 12 UK centers, over the past 20 years shows that the rate of aortic dissection in pregnant women with MFS was 1.9% (one type A and four type B) and there were no deaths. It also reports that pre-conception counseling rates were low [4]. The patient in our case did not undergo pre-conception counseling. If the patient had been diagnosed with MFS before pregnancy, a comprehensive evaluation would have been conducted to determine whether she can be pregnant. According to the 2018 ESC guidelines, pregnancy should be avoided in Marfan patients with an aortic root diameter > 45 mm due to the increased risk of dissection [5]. Earlier studies show that aortic dissection during pregnancy is a life-threatening condition with fetal mortality around 20–30% [6]. Hence, obstetrical specialists are required to show vigilance toward known clinical presentations of MFS, including elongated extremities, wrist and thumb sign, pectus deformity, facial features, scoliosis, and, to facilitate early diagnosis to prevent detrimental outcomes.

To avoid anticoagulant-induced uterine bleeding during aortic surgery, earlier clinicians have conducted hysterectomies as a preventive measure [7, 8]. In the case we present, 20u oxytocin was intravenously administered to the patient after the baby was born. We did not choose intrauterine suture-“B-Lynch” because the wound of “B-Lynch” may bleed during aortic surgery. In order to minimize the wound, we used a Cook balloon to prevent uterine bleeding.

In conclusion, clinicians should pay attention to the possibility of aortic dissection in a pregnant woman with chest, abdominal or back pain because correct and rapid diagnoses and immediate treatment are extremely important. Through this case report, we used Cook balloon during cesarean section to avoid anticoagulant-induced uterine bleeding following aortic surgery in a pregnant patient with Marfan syndrome.

## Data Availability

Not applicable.

## Abbreviations

CTA: Computed tomography angiography
MFS: Marfan syndrome
